# MetaHD: a multivariate meta-analysis model for metabolomics data

**DOI:** 10.1093/bioinformatics/btae470

**Published:** 2024-07-25

**Authors:** Jayamini C Liyanage, Luke Prendergast, Robert Staudte, Alysha M De Livera

**Affiliations:** Mathematics and Statistics, School of Computing, Engineering and Mathematical Sciences, La Trobe University, Kingsbury Dr, VIC 3086, Australia; Mathematics and Statistics, School of Computing, Engineering and Mathematical Sciences, La Trobe University, Kingsbury Dr, VIC 3086, Australia; Mathematics and Statistics, School of Computing, Engineering and Mathematical Sciences, La Trobe University, Kingsbury Dr, VIC 3086, Australia; Mathematics and Statistics, School of Computing, Engineering and Mathematical Sciences, La Trobe University, Kingsbury Dr, VIC 3086, Australia

## Abstract

**Motivation:**

Meta-analysis methods widely used for combining metabolomics data do not account for correlation between metabolites or missing values. Within- and between-study variability are also often overlooked. These can give results with inferior statistical properties, leading to misidentification of biomarkers.

**Results:**

We propose a multivariate meta-analysis model for high-dimensional metabolomics data (MetaHD), which accommodates the correlation between metabolites, within- and between-study variances, and missing values. MetaHD can be used for integrating and collectively analysing individual-level metabolomics data generated from multiple studies as well as for combining summary estimates. We show that MetaHD leads to lower root mean square error compared to the existing approaches. Furthermore, we demonstrate that MetaHD, which exploits the borrowing strength between metabolites, could be particularly useful in the presence of missing data compared with univariate meta-analysis methods, which can return biased estimates in the presence of data missing at random.

**Availability and implementation:**

The MetaHD R package can be downloaded through Comprehensive R Archive Network (CRAN) repository. A detailed vignette with example datasets and code to prepare data and analyses are available on https://bookdown.org/a2delivera/MetaHD/.

## 1 Introduction

Meta-analysis refers to the statistical synthesis of quantitative results from multiple independent studies on a particular research question or hypothesis, with the goal of making inferences about the population effect size of interest (e.g. see Borenstein *et al.* 2009). Statistical meta-analysis methods have emerged as valuable tools in the analysis of metabolomics data (Llambrich *et al.* 2022). In this context of metabolomics data, meta-analysis methods have been used to integrate individual-level data from different cohorts (Tofte *et al.* 2020, Wang *et al.* 2022, Nogal *et al.* 2023) as well as to combine summary estimates obtained from multiple independent studies (Guasch-Ferré *et al.* 2016). The use of a suitable meta-analysis model for integrating individual-level data may also be considered an alternative way of handling unwanted variation commonly encountered in metabolomics studies (De Livera *et al.* 2012, 2015).

Traditionally, meta-analysis methods have combined results from multiple independent studies, each measuring an effect size related to a single outcome of interest (e.g. Borenstein *et al.* 2009). In modern evidence synthesis, particularly in fields like metabolomics, the focus has been on combining results from studies measuring multiple effect sizes associated with correlated outcomes. Thus, the use of multivariate meta-analysis methods that can be implemented within a single modelling framework is of significant value, particularly because the same set of metabolites are not usually measured across multiple metabolomics studies (Wei and Higgins 2013, Sera *et al.* 2019). However, a challenge for researchers is that there are often a large number of metabolites of interest, and hence this high-dimensional setting needs further attention above and beyond what is traditionally considered for multivariate meta-analysis (Jackson *et al.* 2011, Vuckovic *et al.* 2015). Additional challenges include the unavailability of within-study correlations and the parameter estimation of the between-study correlations (Riley 2009, Kirkham *et al.* 2012, Wei and Higgins 2013).

According to recent literature, the most commonly used meta-analysis methods in the context of metabolomics data are: combining fold-changes by logarithmically transforming fold-change values, which are then averaged with weighting by study size (Llambrich *et al.* 2022), *P*-value combinations, one of which is a variant of Fisher’s method (Huo *et al.* 2020, Llambrich *et al.* 2022), using either a fixed-effects model (e.g. Wang *et al.* 2022) or a random-effects meta-analysis model often selected based on a heterogeneity statistic (e.g. Lee *et al.* 2020), or the vote counting approach (e.g. Goveia *et al.* 2016, Llambrich *et al.* 2022, Pu *et al.* 2022). Despite being able to implement with limited data, the fold-change combination approach described above does not take between and within study variability into account, while the p-value combination approach assumes that the distribution of p-values is uniform and does not often account for p-value adjustments which are often made for multiple comparisons in metabolomics studies. The vote counting approach cannot be used to obtain effect sizes and/or confidence intervals. Univariate meta-analysis models, such as fixed-effects or random-effects models, are limited in their ability to model metabolomics data, where the focus is on combining results from studies that have measured multiple effect sizes associated with multiple correlated outcomes. Although being commonly implemented in the metabolomics literature, the use of either a fixed-effects or a random-effects meta-analysis model based on a heterogeneity statistic such as *I*^2^ (Higgins and Thompson 2002), is not a recommended approach in the mainstream statistical literature (von Hippel 2015, Borenstein *et al.* 2017). None of the existing methods mentioned above account for the correlation between the metabolites or missing values. Overlooking these issues when conducting a meta-analysis can yield results with inferior statistical properties such as increases in mean square error (MSE) of parameter estimates, standard error of the final estimates, and bias in the case of non-ignorable missing values (Riley *et al.* 2007, Riley 2009, Wei and Higgins 2013). These can lead to false identification of biomarkers (Type 1 error) or missing out on true biomarkers (Type II error).

In this article, we propose a multivariate meta-analysis model (MetaHD) for integrating and collectively analysing metabolomics data from multiple independent studies, accounting for both within-study and between-study variability. We further account for *within-study correlation*, that is, the correlation due to multiple metabolites being measured from the same observational unit within a study, *between-study correlation* due to the same metabolites measured by multiple studies, and missing values. We show that our approach leads to lower MSE compared to existing methods used for metabolomics data, and is particularly useful in the presence of missing data compared to univariate meta-analysis methods.

This article is organized as follows. In Section 2, we review the existing meta-analysis methods used in the context of metabolomics, and introduce the proposed MetaHD framework together with its estimation approach. In Section 3, using both real and simulated data, we evaluate the proposed MetaHD approach, summarize results, and provide a detailed discussion. In Section 4, we make some concluding remarks.

## 2 Materials and methods

### 2.1 Existing methods for meta-analysis of metabolomics data

We start by summarizing existing meta-analysis methods available for metabolomics data and discussing their limitations in the evidence synthesis of metabolomics data.

#### 2.1.1 Combining fold-changes

For the *i*th metabolite, consider two populations, each with population mean $\mu_{\text{Treated}}^{\left(i\right)}$ and $\mu_{\text{Control}}^{\left(i\right)}$ and population standard deviations $\sigma_{\text{Treated}}^{\left(i\right)}$ and $\sigma_{\text{Control}}^{\left(i\right)}$, respectively. Let ${\bar{x}}_{k,\text{Treated}}^{\left(i\right)}$ and ${\bar{x}}_{k,\text{Control}}^{\left(i\right)}$ denote the two sample mean estimators for the *k*th study with respective sample sizes of $n_{k,\text{Treated}}^{\left(i\right)}$ and $n_{k,\text{Control}}^{\left(i\right)}.$ The combined fold-change is given by


(1) $${\text{FC}}_{\text{Comb}}^{\left(i\right)}=2^{\left(\frac{\sum_{k=1}^{K^{\left(i\right)}}n_{k}^{\left(i\right)}{\;\text{log}\;}_{2}{\text{FC}}_{k}^{\left(i\right)}}{\sum_{k=1}^{K^{\left(i\right)}}n_{k}^{\left(i\right)}}\right)},$$

where,


$$\begin{array}{c} {\text{FC}}_{k}^{\left(i\right)}=\frac{{\bar{x}}_{k,\text{Treated}}^{\left(i\right)}}{{\bar{x}}_{k,\text{Control}}^{\left(i\right)}},\\ n_{k}^{\left(i\right)}=n_{k,\text{Treated}}^{\left(i\right)}+n_{k,\text{Control}}^{\left(i\right)} \end{array}$$

and $K^{\left(i\right)}$ is the number of studies available for the *i*th metabolite (Llambrich *et al.* 2022). While the approach is simple to implement and can be implemented using limited data, the combined fold-change here is only weighted by the sample size. The method ignores variances associated with the individual fold-changes, missing values, and the correlations between the treatment effects.

#### 2.1.2 Combining p-values

The p-value combination approach is widely implemented in metabolomics (Huo *et al.* 2020, Llambrich *et al.* 2022). The p-value $p_{k}^{\left(i\right)}$ in the *k*th study of the *i*th metabolite is transformed in this approach into a Gamma variable ${P^{\prime }}_{k}^{\left(i\right)}=F_{\theta_{k}^{\left(i\right)},2}^{-1}(p_{k}^{\left(i\right)})$ where $F^{-1}$ is the inverse cumulative distribution function of a Gamma variable with the shape parameter $\theta_{k}^{\left(i\right)}=K^{\left(i\right)}\times n_{k}^{\left(i\right)}/\sum_{k=1}^{K^{\left(i\right)}}n_{k}^{\left(i\right)}$, scale parameter 2 and $\sum_{k=1}^{K^{\left(i\right)}}\theta_{k}^{\left(i\right)}=K^{\left(i\right)}$. Then ${\tilde{P}}^{\left(i\right)}=\sum_{k=1}^{K^{\left(i\right)}}{P^{\prime }}_{k}^{\left(i\right)}$ follows a Gamma distribution with the shape and scale parameters of $K^{\left(i\right)}$ and 2 respectively. For observed ${\tilde{p}}^{\left(i\right)}=\sum_{k=1}^{K^{\left(i\right)}}{P^{\prime }}_{k}^{\left(i\right)}$, obtain the combined p-value as,


(2) $${p-\text{value}}_{\text{Comb}}^{\left(i\right)}=P_{\Gamma_{K^{\left(i\right)},2}}({\tilde{P}}^{\left(i\right)}<{\tilde{p}}^{\left(i\right)}),$$

The approach is disjoint from the fold-change approach, and can lead to counter-intuitive results (see Section 3). The approach assumes that the individual p-values are independent and identically distributed, and does not take into account how the p-values have been adjusted in individual studies. Where sufficient summary information is available, the p-value combination approach should not be needed.

#### 2.1.3 A fixed or random effects model based on heterogeneity

For each metabolite, this approach uses either a fixed or a random effects meta-analysis model based on a heterogeneity statistic, with *I*^2^ being the most common statistic used to measure heterogeneity. $I^{2}>40\%$ is often used as a criteria for this selection (see for example, Llambrich *et al.* 2022). However, regardless of the magnitude of the *I*^2^ cut-off or significance based on a p-value, this is not a recommended approach in the mainstream statistical literature (von Hippel 2015, Borenstein *et al.* 2017). Note that the fixed-effects model makes the explicit assumption that there is no heterogeneity, which is often untenable in metabolomics studies due to these studies being conducted in varying conditions such as in different laboratories, different instrumental temperatures, and on different cohorts. Fixed effects models therefore, could result in overly narrower confidence intervals (see e.g. the application presented in Section 3.2). Random effects models which assume that the true effects are different in different cohorts are more justifiable in such settings, and so, despite a low *I*^2^ value (von Hippel 2015, Borenstein *et al.* 2017) the metabolomics studies may have meaningful heterogeneity that warrants a random component in the model.

#### 2.1.4 Vote counting

In this approach, for each metabolite, a value of +1 is allocated for metabolites which are up-regulated, –1 for down-regulated, and 0 for no change, and these counts are then summed for each metabolite (see for example, Llambrich *et al.* 2022). Vote counting does not generate overall effect sizes and associated confidence intervals. Due to this reason, vote counting cannot be directly compared with a specified statistical modelling approach which generates overall effect sizes and confidence intervals. However, to facilitate a comparison with the other statistical models described in the manuscript, we carry out vote counting for all the datasets where the true effect sizes are known, and calculate the proportion of correctly identified changing and non-changing metabolites.

### 2.2 A multivariate meta-analysis model (MetaHD)

To describe the multivariate meta-analysis model (MetaHD), we introduce the following notation.

- Let $Y_{ik}=\text{log}\;\left(\frac{{\bar{x}}_{k,\text{Treated}}^{\left(i\right)}}{{\bar{x}}_{k,\text{Control}}^{\left(i\right)}}\right)$ denote observed (estimated) effects size for the *i*th outcome (metabolite) in the *k*th study, for k=1,…,K and i=1,…,N. Note that some *Y_ik_*s could be missing due to not being reported in some studies. Let $Y_{k}={\left[Y_{1k},Y_{2k},\ldots ,Y_{Nk}\right]}^{⊤}$.
- For the *i*th metabolite, assume that the *population* effect size ${\tilde{\theta }}_{ik}$ in the *k*th study, is drawn from a distribution of *population* effect sizes with *true mean* across the studies *θ_i_* and variance $\sigma_{\theta_{i}}^{2}$. The size of $\sigma_{\theta_{i}}^{2}$ indicates the degree of heterogeneity in the population effect sizes for the *i*th outcome, and *θ_i_* describes their central tendency. Throughout, we let $\theta ={\left[\theta_{1},\theta_{2},\ldots ,\theta_{N}\right]}^{⊤}$.
- Let *τ_ik_* be an error term by which the population effect size ${\tilde{\theta }}_{ik}$ differs from the mean *θ_i_*, representing *true* heterogeneity in effect sizes due to random population effects in the *k*th study. Let $\tau_{k}={\left[\tau_{1k},\tau_{2k},\ldots ,\tau_{Nk}\right]}^{⊤}$.
- Let *ϵ_ik_* represent an error term by which the observed effect size *Y_ik_* differs from ${\tilde{\theta }}_{ik},$ representing the sampling error in the *k*th study. Let $\epsilon_{k}={\left[\epsilon_{1k},\epsilon_{2k},\ldots ,\epsilon_{Nk}\right]}^{⊤}$.
  The MetaHD model is then given by
  (3) $$Y_{k}=\theta +\tau_{k}+\epsilon_{k}.$$

We assume that $\epsilon_{k}∼N(0,S_{k}).$ where $S_{k}$ is a *N* by *N* matrix representing *within-study* variances and covariances of the treatment effects. The off-diagonals of $S_{k}$ reflect the covariation that arises when multiple metabolites are measured on the same observational unit within each study. Further, we assume that $\tau_{k}∼N(0,\Psi )$ where Ψ is a *N* by *N* matrix representing *between-study* variances and covariances of the treatment effects. The off-diagonals of the between-study covariance matrix Ψ reflect the covariation arising when the same metabolites are also measured by other studies. Given $S_{k}$, the estimated values and corresponding standard errors of θ are obtained using the following equations, with some further definition of notations to follow,


$$\begin{array}{c} \hat{\theta }={\left\{\sum_{k=1}^{K}{\left(S_{k}+\tilde{\Psi }\right)}^{-1}\right\}}^{-1}\sum_{k=1}^{K}{\left(S_{k}+\tilde{\Psi }\right)}^{-1}Y_{k},\\ \text{Cov}(\hat{\theta })={\left\{\sum_{k=1}^{K}{\left(S_{k}+\tilde{\Psi }\right)}^{-1}\right\}}^{-1}, \end{array}$$

with $\tilde{\Psi }=\lambda {\hat{\Psi }}^{*}+(1-\lambda )\hat{\Psi }$. Here, ${\hat{\Psi }}^{*}$ is a diagonal matrix obtained using restricted maximum likelihood (Jennrich and Schluchter 1986), $\hat{\Psi }$ is a matrix with diagonal elements equal to those of ${\hat{\Psi }}^{*}$ and the off-diagonals reflecting the covariances estimated using the empirical covariance matrix of $\left[Y_{1},Y_{2},\ldots ,Y_{K}\right]$. Since the number of studies is usually less than the number of metabolites (*K *<* N*) resulting in a singular (non-invertible) estimated covariance matrix of the fold-changes, we use a shrinkage estimator, with the shrinkage parameter, *λ*, is estimated following the data-driven approach described by (Schäfer and Strimmer 2005). When individual-level data are available, we obtain an estimate for $S_{k}$ from the individual-level data, and in other cases we obtain an empirical approximation as described by (Kirkham *et al.* 2012), with the diagonal elements of the *k*th within-study covariance matrix for the *i*th metabolite estimated using


$$\frac{{s^{\left(i\right)}}_{k,\text{Treated}}^{2}}{n_{k,\text{Treated}}^{\left(i\right)}{\bar{x}}_{k,\text{Treated}}^{\left(i\right)}}+\frac{{s^{\left(i\right)}}_{k,\text{Control}}^{2}}{n_{k,\text{Control}}^{\left(i\right)}{\bar{x}}_{k,\text{Control}}^{\left(i\right)}},$$

where ${s^{\left(i\right)}}_{k,\text{Treated}}^{2}$ and ${s^{\left(i\right)}}_{k,\text{Control}}^{2}$ denote sample variances additional to the notation defined earlier.

For model estimation, the missing values in the effect sizes and their associated variances can be handled by allocating a lower weight to the missing outcome. This can be achieved by replacing the missing effect sizes and variances by zero and a large constant value, respectively (Sera *et al.* 2019), so that the missing outcome is allocated a lower weight in the meta-analysis. We found that this approach performed well compared to using other strategies, such as the mean-value imputation method (Steuer *et al.* 2007), the k-nearest neighbour imputation method (Troyanskaya *et al.* 2001), and the eimpute method which uses truncated singular value decomposition (Mazumder *et al.* 2010) (results not shown).

## 3 Results and discussion

We now use three real data examples and a simulation study to demonstrate the performance of the MetaHD methodology, summarize results, and provide a detailed discussion. In our datasets described in Sections 3.1.1 and 3.1.2 where the true effect sizes for the metabolites are known, we compare the methods using root mean square error (RMSE), defined as $\sqrt{E[{\left(\hat{\theta }-\theta \right)}^{2}]}$. In Section 3.2, we also demonstrate the application of the methods using a dataset where the true effect sizes are not known, with the results presented using a forest plot. Following this, in Section 3.3, we carry out a simulation study which, in addition to the RMSE, allows us to further explore both bias and empirical standard error (EmpSE) defined as $E[\hat{\theta }-\theta ]$ and $\sqrt{\text{Var}(\hat{\theta })}$, respectively. In addition to the above, as the vote counting approach cannot provide an overall effect estimate for us to calculate the RMSE, in all datasets where the true effect sizes are known, we calculate the proportion of correctly identified changing and non-changing metabolites and present these using bar plots and box plots.

### 3.1 Combining individual-level data

The datasets analysed in this section are subsets of data extracted from two separate studies in previously published papers (De Livera *et al.* 2012, 2015). Both studies had been designed such that the true effect sizes are known with individual-level data being available. The use of these datasets allowed a direct comparison between the existing methods and the MetaHD approach, by enabling estimated values from each method to be compared with true effect sizes which were known in advance.

#### 3.1.1 Dataset 1

**Figure 1.**
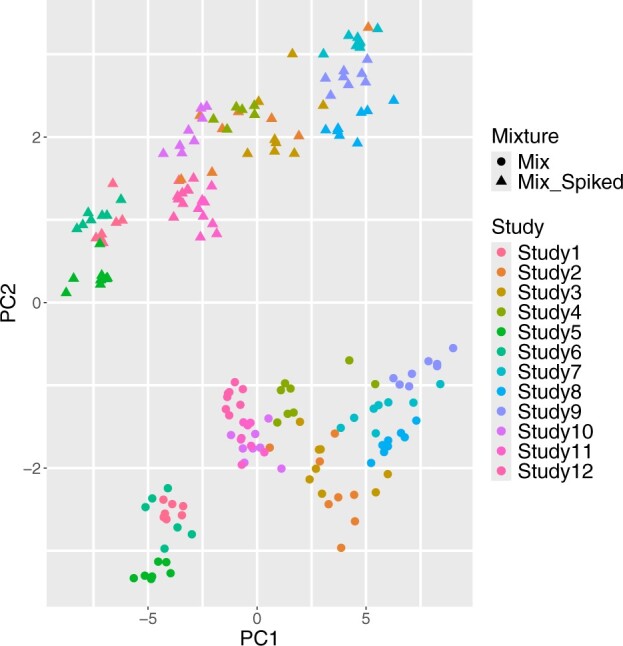
The first two principal components of the whole dataset. There is clear separation due to biological variation, and between-study heterogeneity is also visible.

In this dataset, some replicates of a biological metabolite mixture (MIX) and another set of replicates of the same mixture with some metabolites in increased amounts (MIX-SPIKED) had been run in four different laboratory sites at three different temperature settings on four different gas chromatography-mass spectrometry devices (De Livera *et al.* 2012). Each separate dataset is referred to as ‘study’, and the data used here consist of 32 metabolites and 12 such studies. Eleven metabolites were in 3-fold amounts, and one was in a 5-fold amount relative to MIX, and the other metabolites remained unchanged. The first two principal components of the dataset are shown in Fig. 1. The figure shows that while there is clear separation due to biological variation, there is considerable between-study heterogeneity.

The combined effect estimates for all metabolites were obtained using different meta-analysis methods described in the Section 2 and were compared with the MetaHD approach. Table 1 shows the RMSE values, which give the average of the squared differences between true fold-changes and estimated fold-changes across the metabolites that were non-changing (i.e. true fold-change equal to one) and those that had a 3-fold and 5-fold change.

**Table 1.** Combining individual-level dataset 1: RMSE values on log scale, obtained for the MetaHD approach and other existing meta-analysis methods for metabolomics data.^b^

Method	Non-changing	3-fold	5-fold
MetaHD	**6.8**	**45.1**	**4.6**
Univariate fixed effects model	13.8	50.3	17.7
Univariate fixed/random effects model^a^	10.2	47.2	18.6
Univariate random effects model	10.2	47.2	18.6
Fold-change combination	10.2	48.6	19.7

a Based on heterogeneity.

b Values are in hundreds and the smallest value in each fold-change category is shown in boldface type.

The best-performing technique should generate low RMSE, and the results indicate that the MetaHD approach, which has low RMSE values for all changing and non-changing metabolites, performs comparatively better than the univariate methods and univariate fold-change combination method. The proportion of correctly identified changing and non-changing metabolites and the upset plot showing the shared metabolites identified from each method (shown in Supplementary Figs S1 and S2, respectively) show that the vote counting approach performs poorly compared to other approaches.

#### 3.1.2 Dataset 2

In this experiment, a batch of fetal calf serum had been spiked with a mix of metabolites at different concentration levels (De Livera *et al.* 2012), and three replicate samples had been made at each concentration level. The dataset used here comprised a total of 14 metabolites in two groups, where five of the metabolites had a known 2-fold-change in one group relative to the other, while the rest of the metabolites remained unchanged. The samples in the two groups had been extracted using the same method in a controlled experiment, but run separately in two different instruments: Liquid Chromatography-Mass Spectrometry (LC/MS) and Gas Chromatography-Mass Spectrometry (GC/MS), leading to two separate datasets. Thus, in this example, each separate dataset from each instrument form a different ‘study’ (*K *=* *2), which can be integrated using meta-analysis methods.

The combined effect estimates for all metabolites were obtained using meta-analysis methods described in Section 2.1 and were compared with the MetaHD approach. Table 2 shows the RMSE values on the log scale, obtained from different meta-analysis methods for changing and non-changing metabolites. Here, the values are presented in hundreds.

**Table 2.** Combining individual-level dataset 2: RMSE values on log scale, obtained for the MetaHD approach and other existing meta-analysis methods for metabolomics data.^b^

Method	Non-changing	Changing
MetaHD	**7.6**	**36.8**
Univariate fixed effects model	9.6	51.9
Univariate fixed/random effects model^a^	12.1	42.8
Univariate random effects model	12.1	42.8
Fold-change combination	17.7	43.7

a Based on heterogeneity.

b Values are in hundreds and the smallest value in each fold-change category is shown in boldface type.

The results indicate that the MetaHD has the lowest RMSE values for all changing and non-changing metabolites, which shows the better performance of our approach over univariate meta-analysis methods and fold-change combination approach. The proportion of correctly identified changing and non-changing metabolites and the upset plot showing the shared metabolites identified from each method (shown in Supplementary Figs S1 and S2, respectively) show that the vote counting approach performs poorly compared to other approaches.

### 3.2 Combining summary estimates

The metabolomics dataset analysed herein was obtained from (Llambrich *et al.* 2022) and was originally designed to describe the association of metabolites (amino acid concentration) with lung cancer. Estimates for the means and standard deviations of the *cancer* and *control* groups were available along with the corresponding sample sizes. In this dataset, 21 metabolites were available in six studies. Not all metabolites were present in all six studies, creating some missing values.

**Figure 2.**
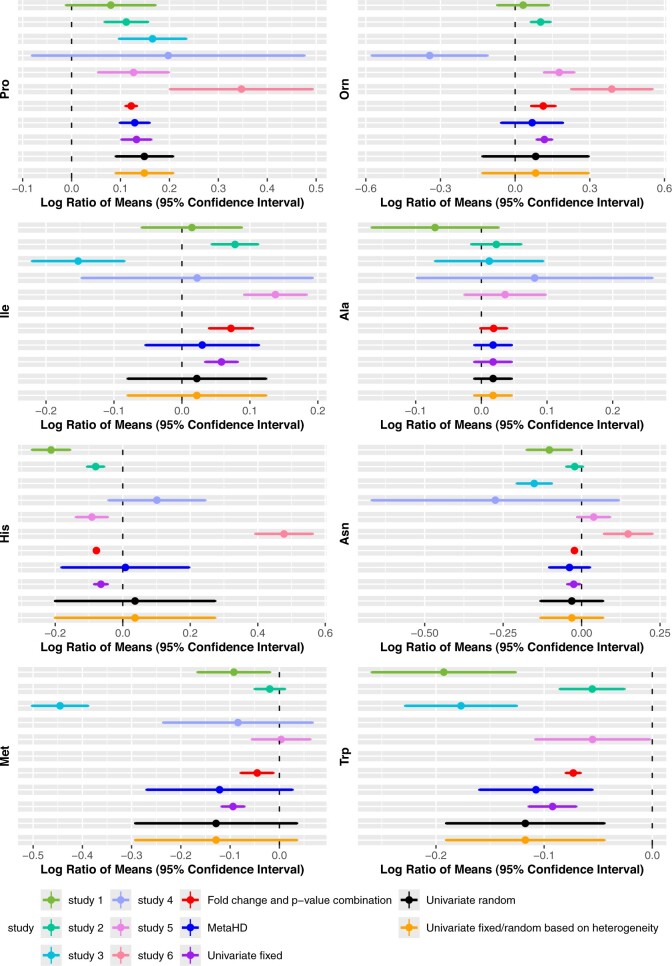
Forest plots showing effect sizes and 95% confidence intervals of individual studies, along with the combined effect estimates obtained from different meta-analysis methods for some selected metabolites.

For all metabolites, we used the fold-change/p-value combination approach, fixed-effects model, random-effects model, fixed-effects/random-effects based on heterogeneity and the MetaHD model to obtain combined effect estimates and standard errors. Vote counting cannot be included in Fig. 2, as it does not generate overall effect sizes and associated confidence intervals. A visual summary of the effect sizes, 95% confidence intervals of individual studies, and those of the combined effect estimates obtained from different meta-analysis methods for some of the selected metabolites are shown in Fig. 2. The figure shows that the fold-change/p-value combination approach (obtained using Equations (1) and (2), respectively) and the fixed-effects model result in overly narrow confidence intervals, leading to conclusions that may not be justifiable given the observed estimates of the individual studies. For example, the results obtained from the above method for Histidine (named ‘His’ in the figure) and ‘Asparagine’ (named ‘Asn’ in the figure) show that the individual results from two to three studies have had minimal effect on the overall result from this method. In contrast, the approaches that include a random-effect component in the model (MetaHD, random-effects model, fixed/random based on heterogeneity) take into account the variability across the studies. Despite the seemingly better performance, as there is no ‘ground truth’ of the values we are trying to estimate for this dataset, in the next section we present an extensive simulation study to further explore the MetaHD approach.

### 3.3 Simulation experiments

We conducted a series of simulation studies to evaluate and compare the MetaHD approach with the existing metabolomics meta-analysis methods. The treatment effects for each study ($Y_{k}={\left[Y_{1k},Y_{2k},\ldots ,Y_{Nk}\right]}^{⊤}$) were generated as $Y_{k}∼\text{MVN}\left({\tilde{\theta }}_{k},S_{k}\right),$ where ${\tilde{\theta }}_{k}∼\text{MVN}\left(\theta ,\Psi \right)$. The unknown parameter values were chosen to mimic those that were obtained from real data: Average within-study variance (log scale)=−1.5, Average between-study variance (log scale)=−5.5, Average between-study correlation=0.25. In doing so, we included both up-regulated and down-regulated metabolites. We further explored the scenarios by changing the within-study correlations by choosing values ranging from low to high at regular intervals.

For each scenario, 1000 meta-analysis data sets were generated and analyzed separately using: (i) MetaHD (estimated): MetaHD model as described in Section 2.2; (ii) Univariate fixed: Univariate fixed-effects model; (iii) Univariate fixed or random: Univariate fixed or random-effects model based on a heterogeneity statistic ($I^{2}>40\%$, a widely-used measure in the field); (iv) Univariate random: Univariate random-effects model; and (v) fold-change and p-value combination method

For comparison purposes, in the figures, we also included the MetaHD model with known covariance structures, as MetaHD (true). To represent the high-dimensional nature of metabolomics data we have encountered in the above examples, we set *N* as 30 and *K* as 12.

Three separate simulation studies were conducted under the above-mentioned scenarios, with (i) one having complete data in all the studies, (ii) one with 5% of the effect sizes and their corresponding variances missing completely at random (MCAR), and (iii) with data missing at random (MAR).

MAR data were generated by specifying a logistic regression model that predicts the probability of missing values given the value of the other metabolites. The logistic model used is: $\text{logit}\left(P(R_{k}=1)\right)=-3+0.9M1_{k}+0.03M2_{k}+1.3M3_{k}-0.4M4_{k}-0.07M5_{k},$ where *R_k_* is a binary indicator that determines whether the metabolite in the *k*th study is missing $\left(R_{k}=1\right)$ or not $\left(R_{k}=0\right)$. We have selected 5 metabolites as missing data predictors (designated as M1, M2, M3, M4, and M5 in the logistic model) and generated missing values in 5 other metabolites on average, with approximately 50%−60% (this can vary depending on the calculated probabilities) of the data being missing in each selected metabolite. This was achieved by generating a binary variable using binomial distribution for each respective metabolite separately, indicating whether the data is missing or not based on the calculated probabilities.

**Figure 3.**
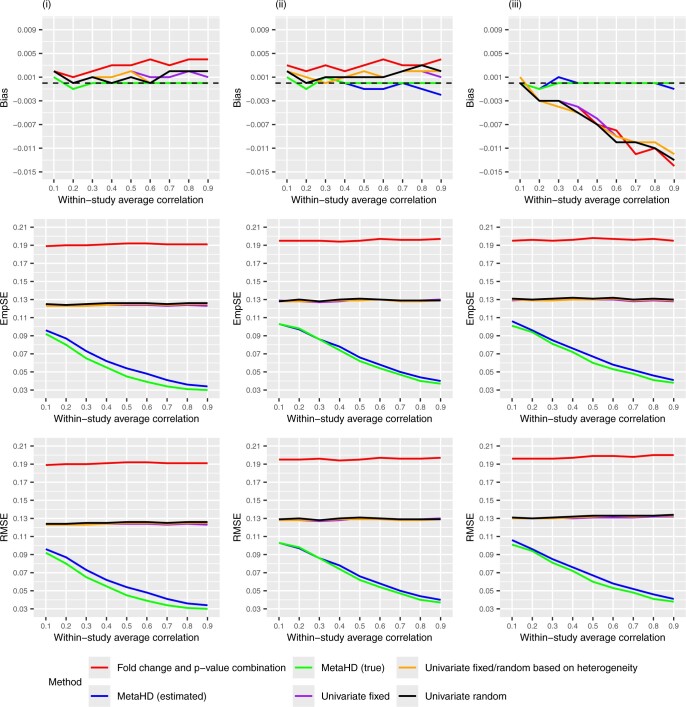
Line plots depicting the bias, EmpSE, and RMSE values obtained from simulation study results for (i) complete data (in the first column), (ii) data MCAR with a 5% missing probability (in the second column), and (iii) MAR data (in the third column).

In simulation scenarios adhering to the MCAR and MAR patterns, we imputed missing values as described in Section 2.2. The bias, EmpSE and RMSE of combined estimates for each outcome were calculated, and the average of these was compared across the different methods. Figure 3 shows the line plots for the bias, EmpSE, and RMSE values obtained from simulation study results for complete data in the first column, data MCAR in the second column and data MAR in the last column.

Figure 3 shows that the MetaHD model performs better than the fold-change combination method and the univariate models by producing approximately unbiased combined estimates with smaller EmpSEs and RMSEs in all three simulation studies conducted. Especially in MAR data simulation scenarios, the univariate models give combined estimates that are biased, with this bias increasing as the within-study correlation increases. However, the MetaHD model was able to considerably reduce this bias by borrowing strength from complete data points.

For each iteration of the simulations, for each method and each scenario described in this article, we calculated the proportion of correctly identified top up-regulated and down-regulated metabolites and presented these using box plots. Box plots showing the proportion of correctly identified top up-regulated and down-regulated metabolites at each iteration of the simulated datasets are shown in Supplementary Fig. S3. While the results are consistent with the conclusions obtained from Fig. 3, it additionally shows that the proportion of correctly identified top metabolites is considerably low for vote counting approach compared with the other approaches.

## 4 Conclusion

In this article, we presented a multivariate meta-analysis approach (MetaHD) for metabolomics data that has several benefits over existing meta-analysis methods. This approach (i) accounts for correlation between metabolites, (ii) considers variability within and between studies, (iii) handles missing values, and (iv) uses shrinkage estimation to allow for high dimensionality (more metabolites than studies).

MetaHD cannot be used with limited data, for example, when only the p-values or only the effect sizes are available. In such cases combining p-values and/or fold changes or vote counting may be the only approaches available. We have compared these approaches in our applications and simulations and we found that out of these, vote counting performed poorly in comparison.

MetaHD uses estimates of the effect sizes, within-study variances, between-study variances and between-study correlations. In addition, MetaHD either estimates or uses available within-study correlations, as explained in Section 2.2. These are also further illustrated in Section 2.2 of the vignette on ‘Preparing the data’ and using the example datasets and the codes presented in detail in Section 3 of the vignette (https://bookdown.org/a2delivera/MetaHD/). It should be noted that both the univariate fixed effects models and univariate random effects models are special cases of MetaHD. For example, when both within and between study correlations are set to zero, MetaHD reduces to a random-effects model and additionally when the between-study variances are set to zero, MetaHD reduces to a fixed-effects model. The example datasets and codes for using MetaHD R package to fit both fixed-effects and random-effects model are also given in the vignette.

The proposed MetaHD model was applied to three real datasets, two of which combine individual-level data, where our approach led to lower RMSEs compared to the existing approaches, and the other that combines summary measures with missing data in some studies. Additionally, extensive simulation experiments that have been conducted under different scenarios have also shown the better performance of the MetaHD approach over existing methods. MetaHD will serve as a valuable tool for integrating and collectively analysing metabolomics data generated from multiple independent studies, facilitating the identification of biomarkers.

## Supplementary Material

btae470_Supplementary_Data
