# Supplementary material for: MetaHD: a multivariate meta-analysis model for metabolomics data
Source: Bioinformatics. 2024 Jul 25;40(7):btae470. doi: 10.1093/bioinformatics/btae470 (PMC11520400; doi:10.1093/bioinformatics/btae470)
Supplement: btae470_Supplementary_Data [file btae470_supplementary_data.pdf]

Supplementary Information for 'MetaHD: A multivariate meta-analysis model for metabolomics data'

Jayamini C. Liyanage [1], Luke Prendergast [1], Robert Staudte [1], and Alysha M. De Livera [1,*]

July 21, 2024

Supplementary Figures

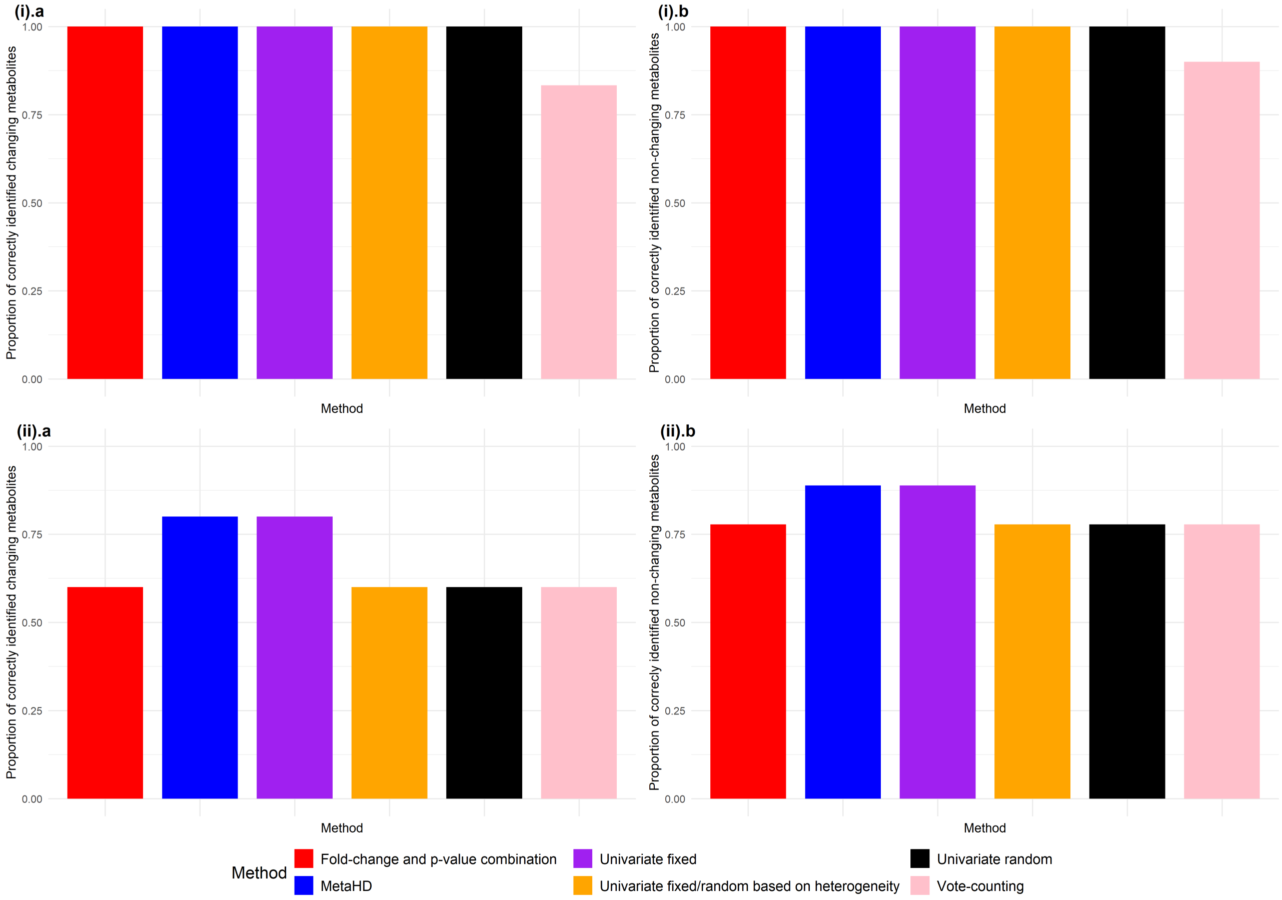

Figure S1: Bar plots showing the proportion of correctly identified changing and non-changing metabolites obtained using the different meta-analysis methods for (i)a: dataset 1, changing metabolites, (i)b: dataset 1, non-changing metabolites , (ii)a: dataset 2, changing metabolites, and (ii)b: dataset 2, non-changing metabolites.

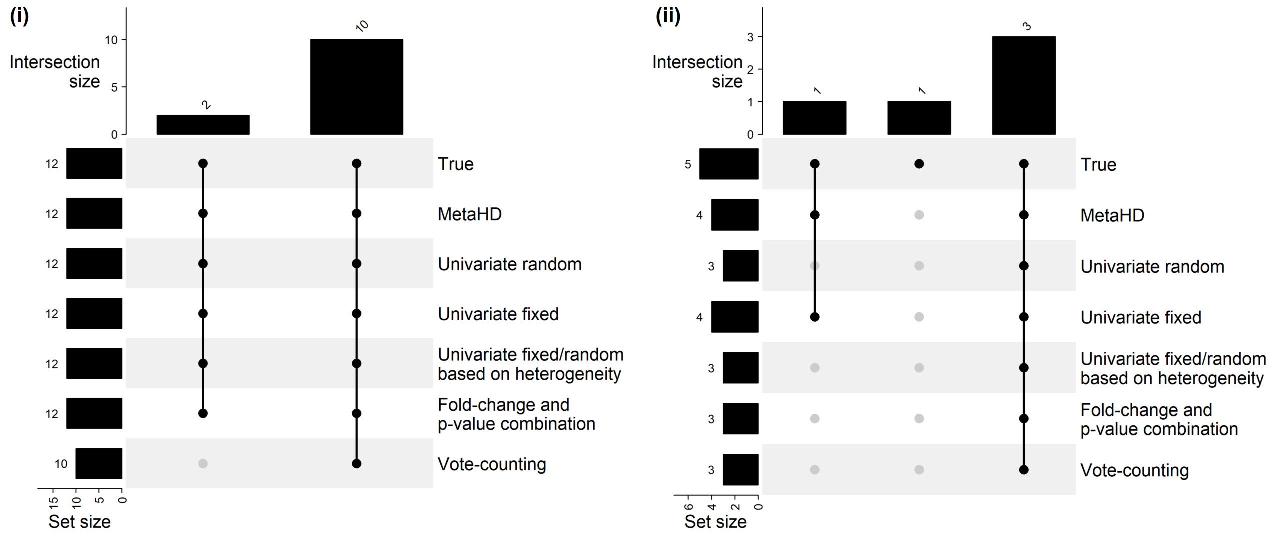

Figure S2: Upset plots showing the shared significant compounds obtained using different meta-analysis methods for(i): dataset 1 and (ii): dataset 2.

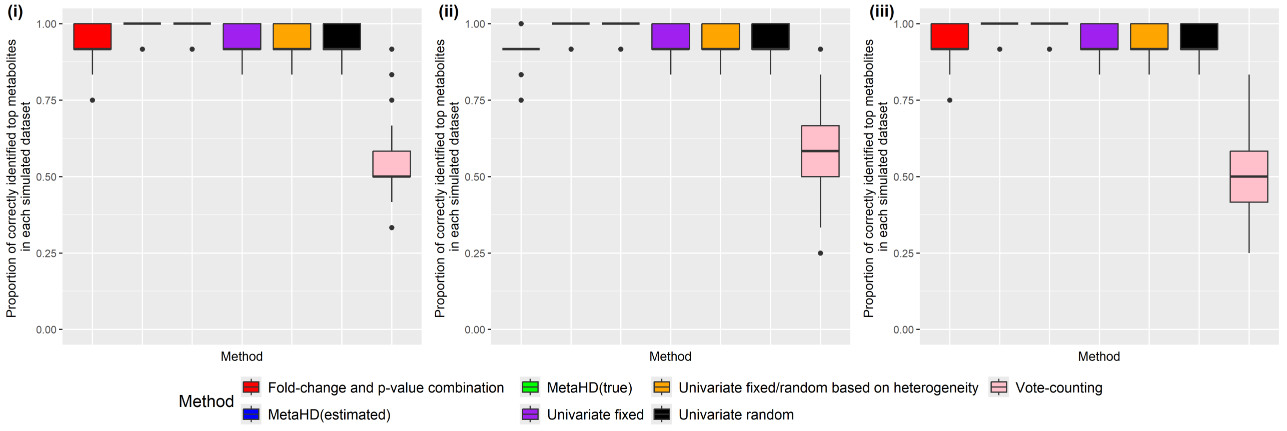

Figure S3: Box plots showing the proportion of correctly identified top twelve up-regulated and down-regulated metabolites at each iteration of the simulated data sets, obtained using different meta-analysis methods for (i): complete, (ii): MCAR, and (iii): MAR data.
